# What is known about the design, delivery and implementation of mental capacity training in care homes?: a scoping review

**DOI:** 10.1186/s12877-025-06476-6

**Published:** 2025-11-07

**Authors:** Nina Jacob, Michelle Maden, Alys Wyn Griffiths, Louis Stokes, Victoria Shepherd, Ruaraidh Hill, Sion Scott, Hayley Prout, Cara Gates, Liz Jones, Lesley Bethell, Peter Hewkin, Grahame Smith, Mishel Ingle, Nefyn Williams

**Affiliations:** 1 Centre for Trials Research, Cardiff University, Cardiff, UK; 2 Liverpool Evidence Synthesis (LENS), Liverpool Reviews and Implementation Group, Institute of Population Health, University of Liverpool, Liverpool, UK; 3 Sheffield Institute for Translational Neuroscience, University of Sheffield, Sheffield, UK; 4 School of Healthcare, University of Leicester, Leicester, UK; 5 Independent Consultant, Sheffield, UK; 6 School of Nursing & Advanced Practice, Liverpool John Moores University, Liverpool, UK

**Keywords:** Mental capacity legislation, Decision making capacity, Nursing homes, Care homes, Training, Education, Dementia

## Abstract

To provide high-quality care to people living in care homes, it is crucial that care home staff possess the knowledge and skills necessary to apply the principles of mental capacity legislation. In order to achieve this, training must be delivered and implemented. This review aimed to identify what is known about the design, delivery and implementation of mental capacity-related education and training in care homes. A scoping review was conducted in line with Joanna Briggs Institute guidance, and reported in accordance with PRISMA-ScR. A total of 3055 records were retrieved by the search and independently screened by two reviewers. A total of 14 papers met the inclusion criteria and were included in the review. Results were synthesised and mapped to the TIDieR checklist. The results highlighted two key factors. Firstly, a standardised one-size-fits-all approach to mental capacity legislation training fails to take account of the diverse needs of both care home residents and staff. Secondly, understanding the relationships between these domains can help overcome barriers and enhance facilitators, leading to more effective training outcomes. The review highlights a knowledge gap, with limited research considering the design and delivery of mental capacity related training. This limits the development of consistent and effective training across the sector. Future research should consider issues of diversity among care home staff or residents, to ensure the appropriateness and applicability of training for all those who receive it.

**Supplementary Information:**

The online version contains supplementary material available at 10.1186/s12877-025-06476-6.

## Introduction

Care homes across the UK provide accommodation alongside personal and/or nursing care [1]. Approximately 75% of older residents living in care homes have moderate to severe dementia or other cognitive impairment, and consequently, many of them will have impaired capacity to make decisions [2–4]. For this reason, it is crucial that care home staff can support residents’ decision-making, ensuring that any decisions made on their behalf are in accordance with underpinning ethical and legal frameworks. This enables them to support residents in making person-centred, ethical and lawful decisions [5, 6].

The Mental Capacity Act (MCA) 2005 [7] provides a legal framework to safeguard the rights of people in England and Wales who lack capacity to make decisions for themselves, whether temporarily or permanently. Equivalent legislation exists in other parts of the UK including the Adults with Incapacity Act (Scotland) 2000 (AWI) [8] and Mental Capacity Act (Northern Ireland) 2016 [9]. The MCA requires professionals to offer appropriate support to adults in their care to make decisions regarding their health, welfare, accommodation, and finances, as well as day-to-day matters, sometimes referred to as “small acts” of care [10].

The MCA Code of Practice (UK Government, 2007) [11] provides guidance on supporting decision-making and assessing capacity, including principles such as ‘best interests’. While the MCA and AWI are primarily used in care homes, the Mental Health Act (MHA) may also be relevant in some circumstances. The MCA and AWI focus on protecting the rights of individuals who lack the capacity to make decisions, whereas the MHA primarily concerns individuals with mental health conditions, regardless of their capacity to consent. In care homes, it is important to recognise that many individuals face issues that could be covered by either framework. This creates a complex legal landscape where both the MCA (and equivalent legislation) and the MHA must be considered when making decisions about care.

In the UK, care home staff include healthcare assistants who typically receive on-the-job training as no formal qualifications are required. In nursing homes, staff work alongside registered nurses to provide a balanced mix of personal care and clinical support. All staff who work with individuals with impaired or reduced capacity should undergo training on mental capacity legislation. However, there is considerable variation in the content and delivery of such training across care homes. Regulatory bodies have raised concerns about the limited understanding of mental capacity within social care, and evidence suggests that implementation of this legislation is lacking in practice [12]. Despite these concerns, there is limited research on best practices for training in mental capacity legislation, and how training approaches vary across the sector remains poorly understood.

Two previous reviews have explored mental capacity training in the UK social care context. The first, in 2013, focused on implementation of the MCA for those working with older adults, but did not address training methods for delivering high quality MCA-compliant care [13]. The second, a 2019 narrative review, evaluated the impact of MCA training on professional practice [14]. This scoping review builds on these by incorporating an additional decade of evidence to comprehensively map current best practices in mental capacity training in the UK care sector.

### Review question

What is known about the design, delivery and implementation of MCA training and education in care homes, including equivalent mental capacity legislation in devolved nations?

## Methods

A scoping review is an appropriate method to (a) identify the scope of available literature on a given topic; (b) provide an overview of concepts relating to the topic; and (c) identify gaps in the literature [15]. Given the limited literature exploring training surrounding the MCA, a scoping review of the available evidence, and evidence gaps, was considered most appropriate. This review was conducted in accordance with the Joanna Briggs Institute guidance for scoping reviews [16] and reported in accordance with PRISMA-ScR (scoping review extension) guidance [17]. The protocol was published on the OSF website [18].

### Search strategy

Searches were conducted in MEDLINE, APA PsycINFO, Embase, CINAHL, Social Care Online, Social Policy and Practice, Health Management Information Consortium (HMIC), Scopus, Google Scholar, LENS.org and the NIHR Journals Library. Additional sources were identified by searching the National Grey Literature Collection (https://allcatsrgrey.org.uk/) and relevant care home organisation websites identified by authors.

An initial comprehensive search strategy using subject headings and free text terms that describe the MCA and care home settings was developed. Search terms were refined with the wider research team and stakeholders, with the search strategy adapted for each database and/or information source (see Supplementary Material 1). Searches were limited to English or Welsh language. In addition, the reference lists of included studies were hand-searched, and citation searches were conducted on the included studies. Search results were downloaded into EndNote 19 and de-duplicated.

### Screening

Using Covidence, two reviewers independently screened all retrieved titles and abstracts against the inclusion criteria. The full texts of selected records were retrieved and assessed against the inclusion criteria by two independent reviewers, with reasons for exclusion documented. Any disagreements regarding inclusion of abstracts or full-text articles were resolved through discussion between the two reviewers, with additional reviewers involved to reach a consensus if necessary.

### Eligibility criteria

#### Participants

Care home staff working with older adults (including care, non-care and management) who are offered or have participated in mental capacity training with assessment, practice or implementation focus or component(s).

##### Exclusions

Staff working in non-social care settings (such as secondary and tertiary hospital healthcare and the community, including peoples’ homes) or who work exclusively with younger adults (i.e. aged 65 or younger) or children.

#### Concept

Training and education programmes covering content around mental capacity legislation and assessment of mental capacity.

#### Context

Care home staff or management based in the UK who are offered or have participated in training with MCA (or equivalent legislation) assessment, practice or implementation focus or component(s). Hospice settings were excluded.

#### Publication types

##### Exclusions

Systematic reviews relevant to the topic were checked for relevant primary studies and then excluded. Studies preceding the date of the MCA 2005 [7] or AWI Act (Scotland) 2000 [8] or MCA (Northern Ireland) 2016 [9] were excluded, dependent on country the training was tailored to.

#### Data abstraction

Two reviewers independently extracted relevant data on participants, concept, context, study methods, and key findings relevant to the review question (e.g., study design, demographics of care setting and overview of training delivered). Additionally, where applicable, components of the design, delivery, and implementation of training were mapped to the Template for Intervention Description and Replication (TIDieR) checklist [19]. Where data were available on the content of the training, this was mapped to the five key principles of the MCA; (1) Presumption of capacity; (2) Support to make a decision; (3) Ability to make unwise decisions; (4) Decisions must be made in the person’s best interest; (5) Least restrictive option should be used.

### Analysis

After familiarisation, one reviewer (NJ) completed preliminary coding of the papers, which was checked by a second reviewer (HP). A codebook was developed and initially mapped to the domains of the TIDieR checklist to support systematic extraction and comparison of intervention descriptions. We also produced a summary table, structured around the TIDieR framework, to present these findings in a clear and accessible format (see Table 1). However, during coding, we noted that while many studies provided considerable detail on aspects related to how well the intervention was implemented (e.g., barriers and facilitators), there was limited information on tailoring, modifications, and particularly on the content of what was actually delivered. As a result, the analysis was extended to include additional inductively generated themes that better reflected the broader patterns emerging from the data.

**Table 1:** TIDieR data extraction

Brief name	Why	What materials	What procedures	Who provided	How	Where	When & how much	Tailoring	Modification	How well (actual & planned)
Alzheimer's Society (2009).	Training resource for people implementing the MCA	Text based quiz to determine existing knowledge of the	Quiz	Aimed at people who have a basic understanding of the MCA	Text based resource	Not reported	Not reported	Training package encourages recipient to reflect and think about how they might feel if a decision was being made for them, and to tailor every decision to the individual	Not reported	Not reported
Care Quality Commission (2012a)	To assess how well providers, commissioners, and staff understand and implement the MCA and DoLS; identify barriers to training and practice; and ensure that care planning, consent, and best interest decisions comply with the legal framework.	- Pocket-sized quick reference guides- Consent and capacity checklists- Safeguarding policies- Booklets and leaflets- Codes of Practice (MCA and MHA)- Information on staff intranet and in handbooks	- MCA/DoLS training sometimes integrated into general induction or unrelated training (e.g., fire safety)- Some organisations had tiered training (e.g., registered staff first, care workers later)- Information dissemination via team meetings, handbooks, and noticeboards- Use of local leads and MCA champions	Varied by setting	- Training delivery methods varied widely: classroom sessions, informal briefings, use of written guides, intranet pages- In some cases, learning was cascaded through staff meetings or led by designated champions	Health and adult social care services across England including care homes, hospitals, and local authority-funded services.	-Highly variable some training only during initial MCA rollout, others offered ongoing or refresher training- Some staff had no MCA/DoLS training at all, while others had attended multiple sessions (e.g., tiered programmes)- Reports of out-of-date or missing training	Some services offered tailored training by role (e.g., managers vs care workers)	Not specified	-Large variation in understanding and implementation- Some staff lacked awareness of basic MCA principles (e.g., confusing MCA with MHA)- Training did not always translate to practice, as evidenced by unreported safeguarding incidents, misuse of DoLS, and misunderstandings around ‘sectioning’ under the MCA
Care Quality Commission (2012b)	To assess implementation of DoLS, with a focus on training levels among care home staff and management, and to monitor uptake and effectiveness of training across the sector.	Training materials not specified in detail; often embedded within broader care topics (e.g. dementia care, safeguarding, consent, human rights)	Training often contextualised within broader care topics. Sometimes delivered initially at DoLS introduction, but not systematically repeated. Some homes had no training or only planned future training.	Not explicitly stated. Training was often recommended by local commissioning teams (e.g. to managers), but delivery agents or educators were not described.	Variable: some formal training sessions (often years out of date), contextual/integrated training with other subjects, or training planned but not yet delivered.	Care homes in England	Timing inconsistent. Some staff had training two or more years prior with no refreshers. Others had not yet attended training. Training generally viewed as a one-time event rather than ongoing professional development	Sometimes targeted by role (e.g. manager vs care worker). Often recommended to senior staff only. No evidence of tailoring to individual learning needs or experience level.	Not specified	Delivery was highly inconsistent. Some homes had trained managers only, while others had not delivered any training. Even when training had occurred, staff often lacked confidence or understanding of DoLS. Emphasis placed on the need for regular refresher training and better tracking (e.g. training matrices not updated). Indicates that even where training occurred, effectiveness and sustained understanding were limited.
Care Quality Commission (2016)	To ensure staff understand and apply the MCA and DoLS properly to protect patients’ rights and promote person-centred care. Address ongoing gaps in training and knowledge to improve quality and safety of care.	Not specified	- Training delivery varies by provider and sector - Use of staff ‘champions’ for mental capacity training in some providers - Documentation and advocacy practices reviewed during inspections - Leadership engagement to support training and culture	Varied across organisations; includes leadership (managers), staff champions, and possibly external trainers. Inspectors note that absence of registered managers often coincided with poor practice.	- Combination of formal training and ongoing staff support - Leadership fostering culture valuing engagement and understanding - Champions supporting peers - Monitoring via CQC inspections	Health trusts, adult social care providers, including nursing and residential care homes across England.	Not specified	Leadership and organisational culture tailored approaches to embed training and understanding. Use of champions to support staff learning adapted to provider needs.	Not specified	Mixed outcomes: many staff demonstrated good understanding of MCA/DoLS, especially in outstanding services - Many services still had inadequate training and understanding - Leadership presence correlated strongly with better training and practice
Davidson et al (2004)	To ensure staff across health and social care understood and could apply Part 5 of the Adults with Incapacity (Scotland) Act (2000), with the goal of improving capacity-related decision-making in practice	Printed materials (47%), discussion guides, PowerPoint presentations, workshops (30%), video (5%) – quality and specificity of materials varied across areas	Varied by area: awareness-raising seminars, full-day specialist training, case-based workshops, team briefings; some areas integrated procedures and guidance into training delivery	Health Board officers, social work departments, some external trainers; occasionally joint NHS–LA teams. Area 1 had a dedicated officer; Area 4 showed a coordinated LA approach. Inconsistent delivery in some regions	In-person group training (lectures, workshops, seminars, awareness sessions); some informal peer learning and ongoing case-based discussions. No remote/online training mentioned	Local authority and NHS Health Board settings across 4 geographic areas of Scotland	Yes – tailored by occupational role (e.g., MHOs, CPNs, psychiatrists, care home staff); informal need-based follow-up training emerged; trainers requested more occupation-specific content	Yes – tailored by occupational role (e.g., MHOs, CPNs, psychiatrists, care home staff); informal need-based follow-up training emerged; trainers requested more occupation-specific content	Ongoing updates encouraged. Recognised need for refresher and follow-up training as legislation evolved and staff experience grew. Some trai`ners hesitant to deliver early due to evolving guidance	Training delivery was highly variable across settings, with significant differences in coordination, content, and uptake. Voluntary participation led to uneven staff engagement, often influenced by trainer reputation and individual enthusiasm. Many staff lacked confidence and requested follow-up or role-specific sessions to support practical application. Key gaps included limited GP involvement and the absence of structured training for private care homes. Barriers such as staffing pressures, fragmented planning, and competing priorities hindered implementation. However, where delivered, training was well-received, and there was widespread recognition of the need for ongoing learning
Gough and Kerlin (2012)	To explore how MCA training is implemented and applied in residential care homes, and to identify barriers and enablers to effective training and application in practice. The study aimed to inform future strategic and operational approaches to training delivery.	Workshops, e-learning, DVD tools, and in some cases real-life case scenario discussions. Suggestions made for additional tools: checklists or crib sheets for supporting decision-making and documenting rationale.	Training delivered primarily through workshops. Some follow-up discussion sessions reported. Managers highlighted the value of in-house, bite-size sessions and workplace-based learning that connects theory with care contexts.	Training developed with support from a sub-regional development group (strategic oversight), local authority, and independent care providers. Delivery often uncoordinated; some care homes had access to dedicated DoLS teams.	Predominantly face-to-face workshops. Some use of digital tools like DVDs or e-learning modules. Limited tailoring to learners' roles. Suggestions included integrated training formats, workplace scenarios, and mixed-method delivery	Training occurred in local authority residential care settings in the East Midlands; often off-site or in workshop environments. Calls for more workplace-based delivery	Not always specified, but training was not mandatory. Delivery was irregular and varied across settings. Follow-up was not routine. High staff turnover disrupted continuity.	Training was not well tailored to job roles or to the care context. MCA was treated as a standalone topic, making it harder for staff to see its relevance. Integration with other care topics recommended	Suggestions included developing flexible delivery options (e.g. bite-size training at shift change), integrating MCA into induction, offering real-life case scenarios, and creating decision-making prompts.	Many managers unaware of how well staff engaged with or applied the training. Some managers misunderstood MCA relevance (e.g. assuming it didn’t apply if residents were assumed to have capacity). Training seen as more effective when supported by e-learning tools and followed by reflective discussions. High demand for better integration, application-based learning, and monitoring tools. Time, cost, and staffing cover remained significant barriers.
Illiffe et al (2015)	To explore implementation and impact of the MCA across four dementia care phases (pre-diagnosis to end of life) in community settings. Specifically:1) Identify implementation issues;2) Make recommendations on CPD and integration with adult safeguarding	Not specified in detail, but includes face-to-face training, supervision, and resources from local MCA/DoLS experts. Staff drew on local safeguarding teams and DoLS professionals as valued resources. Didactic training was commonly used, though often criticised	Longitudinal qualitative interviews with practitioners (n = 272), people with dementia (n = 16), carers (n = 15), and older people (n = 37). Interviews took place at two time points, allowing reflection on training experiences and changes in practice. The study also explored how practitioners learned about the MCA, challenges translating training into practice, and reliance on informal support.	Training providers varied – typically local authorities, safeguarding teams, or specialist MCA/DoLS leads. Staff within local authorities were seen as important sources of support and guidance.	Mixed methods: Mostly didactic training, although this was criticised as ineffective. Very little training occurred through supervision or in practice-based reflective formats. Training delivery was variable and often one-off; gaps existed, particularly in hospital settings and high-turnover environments.	Community-based dementia services including care homes, and healthcare settings across England.	Training was usually one-off; follow-up or refresher training was rare. There was a clear call for continuous, embedded learning processes. Longitudinal design allowed exploration of training over a 9–12 month follow-up period.	Training was not routinely tailored. Didactic formats were often seen as too general. The need for more occupation- and context-specific training was highlighted. The lack of refresher or tiered training also limited practical application.	Not specified, but implications from findings suggest the need to modify training delivery toward ongoing, embedded, and applied models.	Training was inconsistently delivered and variably applied. There were significant concerns about poor uptake in high-turnover environments, lack of hospital-based training, and minimal use of the MCA in supervision. Training was not seen as relevant or welcome when overly abstract. Professionals often relied on informal or overloaded local knowledge sources. Practitioners highlighted the importance of integrating MCA training with broader safeguarding and care practices, and the risks of isolating it as a standalone topic. The need for continuous, experiential, and applied learning was strongly emphasised.
Jayes et al (2022)	To support staff in legally-compliant assessments	Participants used informal (e.g., observation) and formal processes (e.g., the MCA functional capacity test, OPSI 2005, paragraphs 2-3) to assess capacity.	Staff use MCAST guides in formal/informal assessments	All care home staff & qualified nursing staff	Face to face	Five care homes across North West England	Not reported	High – tools and procedures adapted to each resident’s communication needs	Not detailed – no timeline on adjustments made during rollout	Training boosted confidence; audits/CQC noted practice adherence
Manthorpe and Samsi (2009)	To examine how commissioners and practitioners approached the early implementation of the MCA, highlighting strategies, barriers, and variation across two local authority areas. The study emphasised the need for a sustained, multi-method approach to change and ongoing workforce development.	DH-issued scaffolding resources (e.g. implementation guides, PowerPoint slides, IMCA commissioning guidance), SCIE training resources, regional workshops, audit tools, and tailored internal materials (e.g. MCA Leads group notes)	Training included formal sessions (delivered by internal champions or external freelance trainers), policy development, and support structures like MCA Leads Groups. Approaches ranged from isolated training delivery to integrated reflective networks.	MCA Policy Officers, MCA Champions, freelance trainers, MCA Link Officers, supported by Department of Health and SCIE resources.	Combination of face-to-face training, tailored support, peer reflection groups (e.g., Leads Group), and informal discussion	Two local authority case study areas.	Training occurred during MCA roll-out with ongoing support (e.g., LA1’s MCA Leads Group continued two years post-implementation). Scope and frequency varied—some areas had standalone training; others embedded reflective practice.	Tailored support delivered via MCA link officers, training adapted to workforce roles (e.g. learning disability, dementia care); MCA Leads used peer expertise within service settings.	Not explicitly stated, though flexible adaptation to local contexts and professional roles was encouraged.	Observed variation in staff confidence and training impact. Training often seen as one-off event; concern about staff’s ability to translate training into practice due to literacy, language, and learning styles. Highlighted need for multi-method, sustained learning.
Manthorpe et al (2011)	To explore challenges staff face in implementing the MCA, particularly with people with dementia; to assess knowledge, training, expectations, and use of the MCA.	Written literature, email communications, posters, generic presentations, one-day workshops. Staff noted they were inundated with literature but had limited time to engage with it. No specific structured materials used across all homes.	Informal/ad-hoc training practices including peer discussions, notice board information, and incident-based learning. Occasional workshops and presentations; often training seen as one-off events rather than an ongoing process.	Not specified	Mixed delivery modes: written materials, team meetings, posters, informal peer discussion, some one-day workshops. Tended to be reactive rather than structured.	In care homes (no location specified)	Training described as sporadic, event-based, or delivered ‘as needed’. Generally no consistent or comprehensive training programme.	Some tailoring through incident-driven discussions; anticipated introduction of MCA-informed care plans; emerging awareness of IMCA and LPA responsibilities. Evidence of a desire to adapt the Act’s principles to support person-centred care.	Managers anticipated future care plans and documentation to include more MCA guidance. Informal modifications through practical application of principles without structured training.	Knowledge levels varied greatly; confidence often mismatched with actual understanding. Awareness of concepts like IMCA, DoLS, and LPA was limited. Written guidance poorly received. Training perceived as ‘events’, not continuous learning. Despite gaps, staff practices often aligned with MCA values in spirit. Practical understanding often relied on ‘common sense’ rather than formal legal knowledge. Recognition of a need for better integration of law and person-centred care.
Manthorpe et al. (2013)	To support social workers in understanding and applying the MCA, particularly mental capacity assessments—new to their practice—and to address challenges in dementia care and adult safeguarding.	MCA training materials; case law examples; DOLs and Best Interests Assessor training resources; Forms, checklists, and templates for capacity assessment; Peer-led forums and internal discussion materials	Initial one-off MCA training; Advanced DoLS training for some; Informal cascading of training through active case scenarios; Peer discussion and team meetings; MCA explanation to service users in some cases; Manager-initiated MCA forums	- External trainers (MCA, DoLS) - Adult safeguarding leads - Senior practitioners and care home managers - Peer colleagues through team-based learning	In person training sessions - Case-based discussions and reflective practice - Peer forums and team meetings - Informal learning embedded in casework	- Local authority adult social care teams - Care homes - Mental health services - Adult safeguarding settings	- Initial training delivered shortly after MCA implementation (2007–08) - Some received extended training (e.g. one-day sessions, modular format) - DoLS and Best Interests training added later - Peer forums and ongoing informal learning continued beyond initial training	- Case examples used to reflect real life dilemmas -Training most effective when it included debate and practical application - Calls for more depth on concepts like “unwise decisions” - Recommendation for reflective, practice-based training, not purely didactic	Not specified	Basic training valued but insufficient alone - Need for reinforcement and refresher training noted - Application inconsistent; learning through peer support more common than formal supervision - Legal literacy and confidence varied; more advanced training associated with higher competence
Manthorpe et al. (2015)	To explore and improve dementia practitioners’ and safeguarding staff’s understanding and application of the MCA’s criminal offences, enhancing protection against abuse and neglect of people lacking capacity.	- Initial MCA training materials (2007) covering new offences - Case studies from notable prosecutions (e.g., London care home case) - Training presentations including media reports illustrating judicial responses to abuse and neglect	- Initial training on MCA offences at implementation - Ongoing training was lacking or inconsistent - Use of high-profile media cases in training to highlight potential prosecution - Informal sharing of cases among practitioners	Trainers during initial MCA rollout (2007) - Local safeguarding adult coordinators (SACs) sharing knowledge and case examples - Care home managers and dementia practitioners exchanging information informally	Formal training initially; Informal peer discussions and local safeguarding meetings; Use of media case reports as communication tools; Interviews and longitudinal qualitative methods to explore understanding over time	Care homes and dementia care settings in London and SE England	Initial training in 2007. Follow-up interviews and qualitative data collection over 3 years. No consistent ongoing training for new or existing staff noted	Emphasis on raising awareness using media coverage due to lack of formal sustained training	Not specified	- Initial training delivered but not sustained or regularly refreshed - Many newer staff unaware or uninformed about offences - Reliance on media case publicity rather than formal ongoing training limited sustained knowledge and understanding
Manthorpe et al. (2016)	To understand how training, understanding, and practice around the Mental Capacity Act (MCA) evolved in care homes over time, and to assess staff confidence, knowledge, and perceived responsibility. Highlights gaps in legal literacy, the role of organisational structures, and systemic barriers to implementation.	Training materials not clearly remembered by participants; reference to written modules, paperwork in offices (e.g., forms for capacity assessments, LPA info); no reported use of online MCA resources or national material distributed to care homes.	In-house training (usually a single afternoon); some external written modules over months; informal learning through inspection processes, discussions with other professionals, or trade press. Little reference to structured or standardised materials	Not specified	Face-to-face sessions (e.g. in-house afternoons), written self-directed learning modules; largely passive delivery with limited active follow-up or application. No digital or structured online engagement reported.	Training conducted within care homes in South East England	Most training described as one-off, with no ongoing process. Training often dated, forgotten, or minimal. Senior staff more familiar with MCA due to job responsibilities; junior staff generally lacked confidence.	Minimal tailoring reported. Training did not appear adapted to role or level of staff. Participants suggested greater focus was placed on Advance Care Planning (ACP), to the neglect of other aspects of MCA. Training design often missed care assistants’ decision-making roles, legal responsibilities, and practical realities.	Not reported, though the findings suggest that training lacked adaptation or updating over time.	Staff showed variable levels of knowledge and confidence, highly dependent on role and tenure. Managers were most confident; junior staff often unsure or unaware of MCA responsibilities. High staff turnover meant significant attrition in follow-up, highlighting instability in care workforce and risk to sustained implementation. A key barrier was lack of confidence and legal literacy among staff, particularly in relation to ACP and decision-making.
Stanley et al. (2008)	To explore how MCA principles can be supported in everyday care, particularly by using practical tools and approaches to enable communication and support best interest decisions.	Training materials included a Best Interests Checklist to guide practitioners through decisions, prompting reflection on capacity, previous wishes, consultation with others, and documentation.	Not specified; included reflections from practitioners and family carers.	Not specified; Reflections based on co-production of training resources with carers, practitioners, and individuals with lived experience	Training delivered through written materials, case examples, and structured tools like the checklist. Also encouraged reflection and ongoing use of materials in practice.	Not specified – developed for broader training use in health and social care settings.	Not specified. Materials designed for ongoing use, rather than one-off delivery	Strong emphasis on tailoring communication and decision-making support to individual needs, especially for people with non-verbal communication. Encouraged carers to seek and use knowledge from family and others to understand what matters to the person	Not specified	Not reported but tools and checklists were used and valued

We undertook a descriptive-level thematic synthesis, with coding and categorisation focused on identifying common implementation-related issues across studies rather than deep interpretative analysis. This allowed us to group codes into six thematic categories, with discrepancies resolved through discussion. The final themes are presented below and discussed with reference to the TIDieR checklist.

## Results

**Fig. 1:**
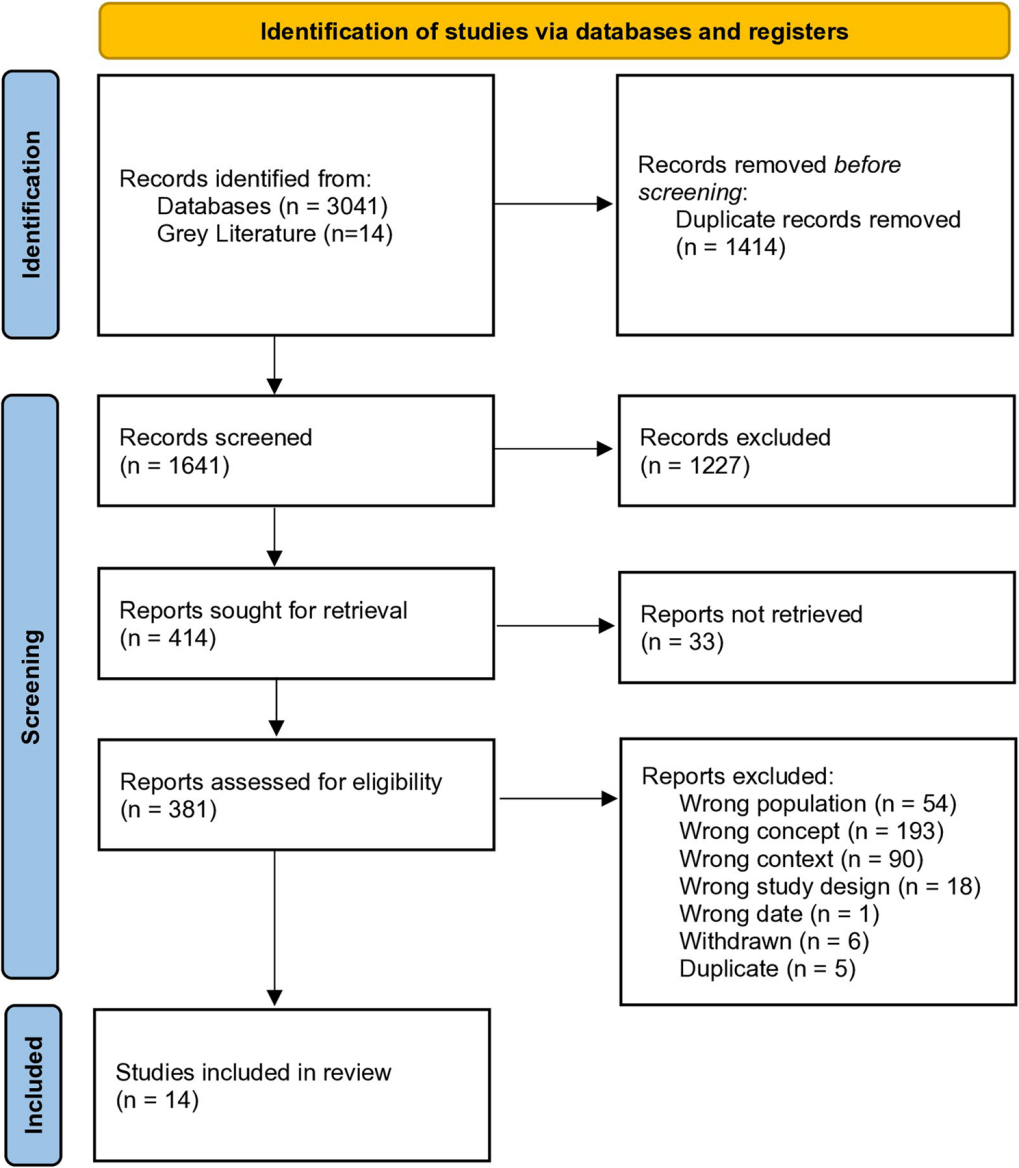
PRISMA flow diagram

The searches identified 3055 potentially eligible records of which, 1414 were duplicates. Following screening of 1641 titles and abstracts, 381 records were retrieved for assessment of the full-text publication. After review of the full-text publications, 14 papers were included in the review (see Fig. 1). Thirteen papers focussed on the MCA (2005) and one paper on the AWI (Scotland) Act 2000. No papers were identified that explored training relating to the Mental Capacity Act (Northern Ireland) 2016. Further characteristics of the included papers are summarised in Table 2. Descriptions of how the training was delivered, specifically the materials used, who provided it, and the mode of delivery was reported by eight papers [20–27]. The content of the training was less frequently reported with six papers [22, 23, 26, 28–30] detailing elements of what was delivered. Thirteen papers reported on barriers and facilitators to the implementation of mental capacity training in care homes.

**Table 2:** Study characteristics

Authors (year)	Location	Approach/methodology	Summary	Included themes
Alzheimer’s Society (2009)	England and Wales	Learning resource and training material	Training resource for people implementing the MCA	Content; Structure;
Care Quality Commission (2012a)	England	Report	CQC report on the monitoring of the deprivation of liberty standards	Content; Structure; Format; Structural factors; Cultural factors; Staff level
Care Quality Commission (2012b)	England	Report	CQC report on how the deprivation of liberty standards were implemented and used	Content; Format; Structural factors; Staff level
Care Quality Commission (2016)	England	Summary of report	CQC reflections on observed practice of implementing DOLs and wider MCA across health and social care	Structural factors; Cultural factors; Staff level
Davidson et al. (2004)	Scotland	Qualitative study	The views of key stakeholders on factors that might facilitate or inhibit the implementation of Part 5 of the Adults with Incapacity (Scotland) 2000	Content; Structure; Format; Structural factors; Cultural factors; Staff level
Gough and Kerlin (2012)	England	Qualitative study	The views of managers of care homes about the delivery, content and implementation of MCA training	Content; Structure; Format; Structural factors; Cultural factors; Staff level
Iliffe et al. (2015)	England	Longitudinal qualitative study	Explore issues relating to implementation of the MCA over a 5 year period with those working with people with dementia and their carers and to make recommendations regarding training and practice	Structure; Format; Structural factors; Cultural factors; Staff level
Jayes et al. (2022)	England	Qualitative study	Identify the challenges & support needed by care home staff around MCA and explore if MCAST toolkit can support decision making	Structure; Cultural factors; Staff level
Manthorpe and Samsi (2009)	England	Case study	Case study of two local authorities looking at the initial roll out, implementation and training of the MCA	Content; Structure; Format; Structural factors; Cultural factors; Staff level
Manthorpe et al. (2011)	England	Qualitative study	Views of care home managers’ and workers’ of knowledge and use of the MCA	Structural factors; Staff level
Manthorpe and Samsi (2013)	England	Longitudinal qualitative study	Views of social work and related practitioners about the expectations, experiences and challenges of implementing the MCA	Content; Format; Staff level
Manthorpe and Samsi (2015)	England	Longitudinal qualitative study	Views and knowledge of dementia care practitioners of the new criminal offences created by the MCA	Content; Structural factors
Manthorpe and Samsi (2016)	England	Longitudinal qualitative study	View and experiences of dementia care staff working in care homes of the MCA five years after it was implemented	Structure; Format; Structural factors; Cultural factors; Staff level
Stanley and Manthorpe (2008)	England	Commentary	Reflections on writing the initial training materials for the MCA	Content; Cultural factors

### Design of mental capacity training

#### Content

Training content was not typically provided, with limited updates since the MCA’s implementation, despite requests for revisions as the Act evolved [21, 26]. A similar pattern was observed with the AWI in Scotland [22]. Manthorpe and Samsi (2009) [25] noted that initial MCA rollout included a series of training guides and information to support implementation, although these are not publicly available. When reported, training typically covered general topics such as understanding capacity, ‘best interests’ decision-making, and the legal aspects of mental capacity legislation [22, 23, 26, 28–30]. However, it was also noted the scope of the MCA made it challenging to cover all elements, resulting in a general overview being dominant [29], rather than detailed information. For instance, the guide produced by Alzheimer’s Society (2009) [28] explains that implementing the MCA involves considering whether someone has the mental capacity to make a specific decision rather than a general assessment of mental capacity and encouraged creative thinking under the fifth statutory principle of the MCA.

Stanley and Manthorpe (2008) [30] suggested that understanding an individual’s perspective requires considering their past wishes, alongside those of their family and friends. Additionally, since the MCA encourages those assessing capacity to review and revise their decisions, training content should focus on equipping staff with key decision-making skills. While various approaches were reported in the papers, a combination of case studies and group discussions was considered the most effective method [23, 25, 28, 30] with participants noting that discussions allowed them to think through elements of capacity and identify where capacity might be misunderstood [25]. Case-studies and practical scenarios were particularly beneficial in helping staff apply theoretical principles in real-world settings, including assessing capacity, determining who should be involved in ‘best interests’ decisions, recording ‘best interests’ consultations, gathering information, and making decisions using the least restrictive options [20, 22, 23, 25, 26, 28–30].

Checklists were also found to be helpful in informing ‘best interest’ decisions [23, 30]. These include considering relevant circumstances, considering whether the decision can be delayed until the person regains capacity (where appropriate), encouraging participation, considering the person’s wishes, feelings, beliefs, and values, and consulting with family and others involved in the person’s welfare. Additionally, checklists emphasised that ‘best interest’ decisions should be informed by in-depth knowledge of the person receiving care or treatment [30].

#### Structure

Eight papers reported on the structure of the training program. An effective training program should adopt a systematic, multi-method approach, integrating formal teaching, informal discussions, and case examples [25]. Two studies emphasized workplace-based training, focussing on real-world scenarios [23, 27. Real-life case studies were valuable for post-training discussions, helping staff apply their learning [23]. Ongoing support can be enhanced through a variety of accessible learning resources, including discussions, share-and-learn approaches, and group activities tailored to staff at all levels and from diverse backgrounds [22, 23, 25]. This multi-method approach reinforces the acquisition of new concepts, deepens practical understanding, and guides decision-making [22].

One study highlighted e-learning’s value for managers, enabling them to monitor staff’s progress and identify areas of weakness [23]. However, a separate study found that staff rarely mentioned online materials, such as those provided to care homes in England to supplement training [27].

Two notable text-based approaches were the MCAST toolkit [31], a paper-based resource that helps professionals document person-centred capacity assessments and support individuals with communication difficulties and an Alzheimer’s Society [28] resource that features quizzes and case studies to assess and test MCA knowledge. These tools can help to evaluate the impact of training on knowledge development, an issue raised by Gough and Kerlin [23].

Conventional workshop-based training was criticised for being too abstract and impractical [23–25]. In contrast, bite-sized training, delivered in small doses during appropriate times, such as shift changes, was valued for its flexibility and cost-effectiveness [23]. A pocket-sized MCA reference guide offered staff quick access to key principles [20],, whilst in Scotland, a consolidated approach with clear guidelines, forms and checklists all integrated into a single, accessible resource was recognised as crucial for effective staff training [22].

### Delivery of mental capacity training

Eight papers discussed the delivery of MCA training. While frequency varied, training was often treated as a ‘one-off event’ [21, 24–27]. Staff received initial training when the Act was first implemented, but this was not updated, leaving new staff without this crucial input [21]. Given the developments in capacity case law, training should reflect these changes and be seen as a continuous process informed by supervision rather than a single event [21, 24, 26]. The distinction between introductory and ongoing training was also emphasised [25]. In Scotland, ‘next stage’ training including follow-up and refresher courses was deemed essential. Ultimately, training should be continuous, supporting staff to apply new information [21].

There was no consensus on the format of training. Some argued it should be delivered in isolation and not combined with other exercises [21], while others suggested it should be integrated with topics relevant to daily care-home work, such as dementia care and managing challenging behaviour [22, 23]. It was further highlighted that training in social care typically involves integrating new knowledge into daily work rather than merely imparting information [24–26].

### Implementation of mental capacity training

Of the 13 studies that discussed the implementation of MCA training, it was largely from the perspective of the barriers and facilitators to effective implementation. Our synthesis identified two overarching categories of barriers or facilitators: system domains and staffing domains.

#### System domains

These relate to the overarching *structure* and *culture* of a care home organisation, including how it interacts with its environment. System domains focus on macro-level factors influencing how MCA training is implemented and embedded into a care home.

#### Staff domains

These focus on individual care home staff, including the skills, ability and confidence of staff members. This domain examines how individual factors influence the implementation and success of MCA training.

### System level barriers and facilitators

#### Structural factors

Key barriers identified in ten papers included high workload, staff turnover and shortages, the cost and time involved in delivering training and competing priorities. The most common issues across all nations were staff shortages, high turnover, and the large number of staff requiring training [12, 20–24, 29, 32]. High staff turnover made it difficult to monitor training, and managers noted the challenge of releasing staff for training, especially in smaller care homes with smaller budgets and fewer staff to provide cover [12, 23].

Differences in training were observed between different forms of provider, with some reports indicating that private care homes often lacked responsibility for providing training [24]. Consequently, a lack of training was routinely reported across studies, with responsibility falling on a small number of staff most committed to capacity legislation, rather than generating change at an institutional level [12, 20–23, 25, 27, 32]. In Scotland, staff training on the AWI was not mandatory and consequently level and uptake of training depended largely on the trainers’ enthusiasm and reputation [22].

Facilitators to address these issues included investing in a dedicated team to deliver training [20, 22, 23] and designating staff with responsibility for safeguarding training within each organisation [12, 25, 26, 32]. In-house training was also a considered cost-effective solution [25].

To foster change at the institutional level, Commissioners can gain insights into local activity by developing or using existing audit tools [25]. Such tools would help measure and review provision of training, assess compliance with the MCA, and serve as benchmarks for improvements. Commissioners could also monitor indicators of partial or patchy training, such as complaints, adult safeguarding referrals, serious case reviews, and the reports of Court of Protection visitors.

#### Cultural factors

Barriers and facilitators related to workplace culture, particularly concerning system-level commitment to training on the both the MCA and AWI, as well as organisational readiness for change, were identified in nine papers. Despite challenges posed by high staff turnover, there was an appetite for training among employees, observed across all nations [22, 25]. However, some managers regarded the training as irrelevant to their care home, indicating a lack of awareness of the MCA’s relevance to their roles [23, 24]. Additionally, there was evidence that the MCA was not routinely used during staff supervision, highlighting the need for its integration into line management and supervision meetings [24].

Cultural barriers also included a lack of trust in trainers [24]. The absence of a designated person responsible for delivering training and unfamiliarity with trainers affected the uptake of training [32]. Addressing these issues requires building trust with trainers, ensuring a dedicated team is responsible for training, and making the relevance of the MCA clear to all staff, including managers [20, 27, 30]. Strong links between Independent Mental Capacity Advocates (IMCAs) and care homes were also found to help cascade knowledge, with IMCAs taking on some responsibility for training in certain care homes [25].

Several studies highlighted the value of having ‘joined up’ training between health and social care to address barriers around workplace culture [22, 31]. In Scotland, an integrated approach, where training is delivered in partnership between NHS trusts and social care/social work was seen as beneficial. Improved communication between social care and healthcare staff may facilitate partnership working across professional boundaries, enabling the delivery of holistic, seamless care [22].

### Staff level barriers and facilitators

While system domains focused on the overall structure and culture, staff domains focus on individual experiences, reflecting on the training received and their confidence in translating mental capacity legislation into practice. This distinction was discussed in 11 papers. Notably senior staff and managers were more likely to receive training, but there was no formal system in place to ensure this knowledge was cascaded to all staffing levels [12, 20, 21, 26]. There was also confusion among junior staff regarding the differences between the Mental Health Act (MHA) and MCA [20].

Training received by junior staff often failed to translate effectively into practice [20]. Many lacked confidence in applying the MCA, although they felt they could consult someone for urgent or difficult issues [12, 24, 25, 27]. Some were reluctant to take responsibility for decisions under the MCA, highlighting a lack of confidence and legal literacy [27]. There were also concerns about staff’s ability to learn from written materials, potentially due to literacy and language barriers [25, 27]. Additionally, uncertainty about adult learning principles and limited experience with self-directed learning were noted [25]. Trainers may also face demands for traditional classroom-based training methods, as managers may favour these approaches due to limited exposure to other models of self-directed learning [25].

More experienced senior staff talked of their familiarity with the MCA and its provisions [12, 27]. Not surprisingly, managers were the most confident in their knowledge of the MCA, which they described as stemming from discussions with other professionals and inspection requirements, as well as information gleaned from training [12, 27].

Interestingly, it was reported that staff who had not received training under the AWI (Scotland) created additional challenges for those who had [22]. Additionally, a lack of training among healthcare professionals, particularly GPs, made communication between health and social care more challenging [22]. To address these issues, facilitators suggested that training should be customised to the specific roles of staff and follow a tiered approach, where information is tailored to an individual’s role rather than providing a general overview of the capacity legislation [20, 22, 23, 25]. At the same time, this approach should maintain a common language across training to facilitate better and more effective learning [25]. Such training would promote collaborative, interdisciplinary practices as best practice, whilst recognising that current UK legislation does not specify which disciplines or seniority levels should assess mental capacity [31].

## Discussion

We aimed to review the existing literature of the design, delivery and implementation of MCA (or equivalent legislation) training within care homes and the barriers and facilitators that support this. This review highlights two important factors. Firstly, a blanket approach to training is not sufficient for care homes; key domains must be addressed to ensure more effective implementation of training in practice. Secondly, understanding how these different domains interact can help inform more comprehensive strategies to reduce barriers and strengthen the factors that support successful implementation. When staff lack understanding or fail to implement the MCA appropriately, residents may lose opportunities to make everyday choices, undermining their autonomy and the Act’s principles. As noted in the introduction, supporting “small acts” of care is crucial - without this, residents risk disempowerment and a diminished sense of control over their daily lives.

This review found that a standardised one-size-fits-all approach to mental capacity legislation training fails to take account of the diverse needs of care home residents and staff. Care homes vary in terms of staff expertise, residents’ needs and organisational priorities; consequently, training on mental capacity legislation should be tailored to meet these unique issues. There was limited agreement within the literature on the most appropriate format or frequency of training. While various approaches were reported in the papers, a combination of case studies and group discussions was considered the most effective method for strengthening the application of the key principles of mental capacity legislation. Staff reflections of e-learning were often negative [27]. This aligns with existing research considering optimal ways to provide dementia awareness training to social care staff, which found that staff generally dislike online-only formats, favouring face-to-face training that include practical application relevant to their daily work, such as demonstrating examples of good or poor practice [33].

Differences in training format remain, with some advocating for standalone training and others for it its integration into broader topics relevant to daily care-home work, such as managing challenging behaviour or promoting resident’s rights. These discrepancies likely reflect variations in staff role, care home environment or organisational culture, highlighting the need for further research to understand their impact on training effectiveness.

Newly appointed NHS healthcare staff often have patchy knowledge and lack baseline knowledge of the MCA, highlighting the importance of early training on mental capacity legislation [34]. This is crucial as care staff often defer decisions to colleagues, suggesting limited confidence and awareness of their responsibilities under the MCA [35]. Those entering the role may have less prior care experience and shorter inductions than those working in healthcare. Addressing these issues is essential in a sector where hierarchical structures are prominent and junior staff are unlikely to challenge more senior staff [36].

It is also worth noting that the lack of training among healthcare staff impacts social care, with one study reporting a notable gap in training for GPs on the AWI in Scotland. However, this could be attributed to Scotland’s more integrative approach to mental capacity legislation and training, where the AWI explicitly encourages multi-disciplinary collaboration across health, legal and social services. In Scotland, the emphasis is on joint training of professionals from multiple sectors, whereas the MCA tends to focus more narrowly on decision making processes, particularly in assessments of capacity for specific decisions. Consequently, at the time of publication of the one paper exploring AWI, the lack of training in certain roles or sectors was more apparent in Scotland, where collaborative working is more heavily emphasised. Concerns about the gaps in knowledge of the AWI across the health, social work and social care workforce has led to a new national approach to learning and training on applying the requirements of the AWI [37].

The review also identified additional domains – such as staff engagement, content relevance and organisational factors– and how these might be utilised to overcome barriers to training. For instance, the lack of training and awareness of MCA relevance to staff roles emerged as a key barrier. High turnover of a staff, a common characteristic of many care homes, exacerbates this issue, emphasising the need for regular, in-house training. Repeated introductory training, along with more advanced experiential-focused courses, were deemed necessary to enhance learning and consolidate its application in real-world care. Understanding the feasibility of implementing this model of training should be undertaken.

The review also shed light on significant operational barriers to MCA training, particularly the lack of time and availability of senior support. In additional to the hierarchical issues identified above, the challenges within both for-profit and not-for-profit care homes may lead to limited budgets or resource constraints which can deprioritise training. To address these challenges, it is important to embed MCA training and learning from this into the daily routines of the care home, such as incorporating it into clinical supervision, team meetings and ongoing professional development. This approach would encourage staff to robustly and continuously reflect on their practice learning with the aim of improving the care they deliver. Additionally, the role of leadership is crucial, without visible and consistent support from senior managers the nuanced and considered approach that is needed for MCA training is unlikely to be fully realised.

The training of staff, the delivery of person-centred care practices, and the assessment of decision-making capacity in older adults are a global concern, particularly in residential care settings [38]. Ireland’s Assisted Decision-Making (Capacity) Act 2015 reflects a broader international shift toward functional, right-based approaches to capacity assessment, as observed in not only the UK’s legislation but also Canada and Australia [39] However, implementation challenges persist. International evidence highlights gaps in training in practice. In India, limited protocols contribute to inconsistent assessments in dementia care [40], while UK healthcare professionals report uncertainty in assessing capacity among people due to insufficient guidance. These findings underscore the need for well-designed, practical capacity training opportunities in care homes that align with legal and ethical standards.

Statutory differences exist between the legal frameworks governing mental capacity [41], and the evolving implementation of related training. Although the MCA has been in place for over 15 years and the AWI in Scotland even longer, training has not been updated to reflect the evolving understanding and case law. This gap will likely be exacerbated with the transition from the Deprivation of Liberty Standards (DOLs) to the Liberty Protection Standards (LPS) and the updated *Code of Practice* as outlined in the CQC’s *State of Care* report (2022–2023) [42]. Similarly, the AWI Act has been under long-standing consultation in Scotland, reflecting ongoing efforts to modernise the legislation to better align with current needs and practices. In Northern Ireland, the MCA has only recently been implemented, so it is arguably too early to assess its full impact. Nonetheless, as legal frameworks evolve, training must adapt to ensure that all staff are adequately prepared to apply mental capacity legislation in practice.

The findings of the present review focus on the design and delivery of training, which are complemented by our systematic review focusing on the barriers and facilitators to the practical implementation of mental capacity legislation [43] which outlines what supports and hinders such legislation being enacted within residential care homes in the UK.

### Limitations and future directions

This review focused on the design and delivery of mental capacity training in the UK context, meaning that the implementation of such legislation in other geographies is not explored. The review identifies a gap in the literature; despite the MCA being in place for nearly two decades and the AWI even longer, only thirteen papers focussing on training related to mental capacity legislation in care homes were identified. Limited evidence exists on both the content and development of training, highlighting a critical issue: the lack of knowledge sharing between organisations. This lack of cross-organisation collaboration may hinder the development of consistent and effective training across the sector, negatively impacting care quality.

None of the included papers considered staff or resident diversity. As the population ages, and the demand for professional care increases, the social care sector increasingly relies on more ethnically diverse teams, often including immigrant workers [44]. Having a diverse staff team, in terms of gender, ethnicity, sexual orientation or age, is known to improve quality of care [45], and therefore, considering the role of culture within training and implementation of mental capacity legislation is crucial, and currently remains unexplored.

A lack of training for external healthcare professionals, including GPs, highlights systemic issues beyond care homes, where there is no current ‘usual model’ across organisations. Future research should explore inter-professional training models, where care home staff and healthcare workers receive joint training and different roles and responsibilities are outlined. This could help clarify roles, address concerns around hierarchical structures and build confidence in challenging poor practice. Additionally, research should seek to understand the optimal strategies of training social care staff about mental capacity legislation and how to support the translation of this learning into practice.

## Conclusion

This systematically based scoping review demonstrates significant inconsistencies in the delivery and implementation of mental capacity training within care homes in the UK. These inconsistencies are likely to be exacerbated by a lack of shared leaning across the sector, with each organisation developing its own approach to training, often delivered in isolation. To ensure that mental capacity training is not only consistent in quality but also effective, there is a need to develop standardised frameworks that can be adapted to the specific needs of an individual care home, with the overall aim of embedding training and learning into everyday practice of the care home. Further research should establish an in-depth understanding of MCA training across England and Wales, to begin to unpick the current state of training and develop recommendations for improving content, delivery and implementation within the sector.

## Supplementary Information

Supplementary Material 1.

## Data Availability

Data sharing is not applicable to this article as no datasets were generated or analysed.

## References

[1] Gordon AL, Franklin M, Bradshaw L, Logan P, Elliott R, Gladman JRF. Health status of UK care home residents: a cohort study. Age Ageing. 2014;43(1):97–103. 10.1093/ageing/aft077. 23864424 10.1093/ageing/aft077 PMC3861334

[2] Hood K, Nuttall J, Gillespie D, Shepherd V, Wood F, Duncan D, et al. Probiotics for antibiotic-associated diarrhoea (PAAD): a prospective observational study of antibiotic-associated diarrhoea (including clostridium difficile-associated diarrhoea) in care homes. Health Technol Assess. 2014;18(63). 10.3310/hta18630. 10.3310/hta18630 PMC4781053 25331573

[3] Stewart R, Hotopf M, Dewey M, Ballard C, Bisla J, Calem M, et al. Current prevalence of dementia, depression and behavioural problems in the older adult care home sector: the South East London care home survey. Age Ageing. 2014;43(4):562–7. 10.1093/ageing/afu062. 24855111 10.1093/ageing/afu062

[4] Wade DT. Determining whether someone has mental capacity to make a decision: clinical guidance based on a review of the evidence. Clin Rehabil. 2019;33(10):1561–70. 10.1177/0269215519853013. 31169035 10.1177/0269215519853013

[5] Care Quality Commission. Monitoring the use of the mental capacity act deprivation of liberty safeguards in 2012/13. Newcastle upon Tyne: CQC; 2014.

[6] Manthorpe J, Harris J, Samsi K, Moriarty J. Doing, being and becoming a valued care worker: user and family carer views. Ethics Soc Welf. 2016;11(1):79–91. 10.1080/17496535.2016.1247904.

[7] Department of Health. The mental capacity act. London: HMSO; 2005.

[8] Adults with Incapacity. Scotland) act 2000. Scottish Executive; 2000.

[9] Department of Health and Social Services and Public Safety. Northern Ireland. Mental Capacity Act (Northern Ireland). 2016.

[10] Samsi K, Manthorpe J. Everyday decision-making in dementia: findings from a longitudinal interview study of people with dementia and family carers. Int Psychogeriatr. 2013;25(6):949–61. 10.1017/S1041610213000306. 23510662 10.1017/S1041610213000306

[11] Department for Constitutional Affairs. Mental capacity act 2005: code of practice. London: The Stationery Office; 2007.

[12] Manthorpe J, Samsi K, Heath H, Charles N. Early days’: knowledge and use of the mental capacity act 2005 by care home managers and staff. Dementia. 2011;10(3):283–98. 10.1177/1471301211403970.

[13] Hinsliff-Smith K, Feakes R, Whitworth G, Seymour J, Moghaddam N, Dening T, et al. What do we know about the application of the mental capacity act (2005) in healthcare practice regarding decision-making for frail and older people? A systematic literature review. Health Soc Care Community. 2017;25(2):295–308. 10.1111/hsc.12310. 26611194 10.1111/hsc.12310

[14] Jenkins C, Webster N, Smythe A, Cowdell F. What is the nature of mental capacity act training and how do health and social care practitioners change their practice post-training? A narrative review. J Clin Nurs. 2020;29(13–14):2093–106. 10.1111/jocn.15256. 32223040 10.1111/jocn.15256

[15] Munn Z, Peters MDJ, Stern C, et al. Systematic review or scoping review? Guidance for authors when choosing between a systematic or scoping review approach. BMC Med Res Methodol. 2018;18:143. 10.1186/s12874-018-0611-x. 30453902 10.1186/s12874-018-0611-x PMC6245623

[16] Peters M, Godfrey C, McInerney P, Munn Z, Tricco A, Khalil H. Chapter 11: scoping reviews (2020 version). In: Aromataris E, Munn Z, editors. JBI manual for evidence synthesis. JBI; 2020.

[17] Tricco AC, Lillie E, Zarin W, O’Brien KK, Colquhoun H, Levac D, et al. PRISMA extension for scoping reviews (PRISMA-ScR): checklist and explanation. Ann Intern Med. 2018;169(7):467–73. 10.7326/M18-0850. 30178033 10.7326/M18-0850

[18] Maden M, Griffiths A, Scott S, Gates C, Stokes L, Hill R et al. Scoping review of best practice in mental capacity act training in care homes (ENACT study). 2024. 10.17605/OSF.IO/CP9AT

[19] Hoffmann TC, Glasziou PP, Boutron I, Milne R, Perera R, Moher D, et al. Better reporting of interventions: template for intervention description and replication (TIDieR) checklist and guide. BMJ. 2014;348:g1687. 10.1136/bmj.g1687. 24609605 10.1136/bmj.g1687

[20] Care Quality Commission. Monitoring the use of the mental capacity act deprivation of liberty safeguards in 2011/12. Newcastle upon Tyne: CQC; 2012a.

[21] Care Quality Commission. The operation of the deprivation of liberty safeguards in England, 2010/11. Newcastle upon Tyne: CQC; 2012b.

[22] Davidson S, Wilkinson H, Urquhart G, Wasoff F, Mason A. Review of the implementation of part 5 of the adults with incapacity (Scotland) act 2000: A qualitative study of implementation and early operation. Edinburgh: Scottish Executive Social Research; 2004.

[23] Gough M, Kerlin L. Limits of mental capacity act training for residential care homes. J Adult Prot. 2012;14(6):271–9. 10.1108/14668201211286048.

[24] Illiffe S, Wilcock J, Drennan V, Goodman C, Griffin M, Knapp M, et al. Changing practice in dementia care in the community: developing and testing evidence-based interventions, from timely diagnosis to end of life. Prog Grants Appl Res. 2015;3(3). 10.3310/pgfar03030. 25950075

[25] Manthorpe J, Samsi K. Implementing the MCA 2005: challenges for commissioners. J Integr Care. 2009;17(3):39–47. 10.1108/14769018200900023.

[26] Manthorpe J, Samsi K. Changing practice: adapting to the mental capacity act 2005. Soc Care Neurodisabil. 2013;4(3/4):124–33. 10.1108/SCN-03-2013-0008.

[27] Manthorpe J, Samsi K. Care homes and the mental capacity act 2005: changes in Understanding and practice over time. Dementia. 2016;15(4):858–71. 10.1177/1471301214542623. 25015949 10.1177/1471301214542623

[28] Alzheimer’s Society. Supporting people with dementia using the mental capacity act. London: Alzheimer’s Society; 2009.

[29] Manthorpe J, Samsi K. Care professionals’ Understanding of the new criminal offences created by the mental capacity act 2005. Int J Geriatr Psychiatry. 2015;30:384–92. 10.1002/gps.4147. 24890855 10.1002/gps.4147

[30] Stanley N, Manthorpe J. Small acts of care: exploring the potential impact of the mental capacity act 2005 on day-to-day support. Soc Policy Soc. 2008;8(1):37–48. 10.1017/S1474746408004569.

[31] Jayes M, Austin L, Brown L. Supported decision making and mental capacity assessment in care homes: a qualitative study. Health Soc Care Community. 2022;30(4):e1061–9. 10.1111/hsc.13512. 34250675 10.1111/hsc.13512

[32] Care Quality Commission. The state of health care and adult social care in England 2015/16. Newcastle upon Tyne: CQC; 2016.

[33] Surr CA, Gates C, Irving D, Oyebode J, Smith SJ, Parveen S, et al. Effective dementia education and training for the health and social care workforce: a systematic review of the literature. Rev Educ Res. 2017;87(5):966–1002. 10.3102/0034654317723305. 28989194 10.3102/0034654317723305 PMC5613811

[34] Willner P, Bridle J, Dymond S, Lewis G. What do newly appointed health staff know about the mental capacity act (2005)? Med Sci Law. 2011;51(2):97–101. 10.1258/msl.2011.010120. 21793472 10.1258/msl.2011.010120

[35] Manthorpe J, Samsi K, Rapaport J. Dementia nurses’ experience of the mental capacity act 2005: a follow up study. Dementia. 2014;13(1):131–43. 10.1177/1471301212454354. 24381044 10.1177/1471301212454354

[36] Essex R, Dillard-Wright J, Aitchison G, et al. Everyday resistance in the U.K.’s National health service. Bioeth Inq. 2023;20:511–21. 10.1007/s11673-023-10274-3. 10.1007/s11673-023-10274-3 PMC10624704 37713010

[37] Mental Welfare Commission for Scotland. (2023, June 8). New learning site launched on Scotland’s Adults with Incapacity law. https://www.mwcscot.org.uk/news/new-learning-site-launched-scotlands-adults-incapacity-law

[38] Usher R, Stapleton T. Assessment of older adults’ decision-making capacity in relation to independent living: A scoping review. Health Soc Care Community. 2022;30(6):e2745–57. 10.1111/hsc.13487. 10.1111/hsc.13487 34288195

[39] Browning M, Bigby C, Douglas J. A process of decision-making support: exploring supported decision-making practice in Canada. J Intellect Dev Disabil. 2020;46(2):1–12. 10.3109/13668250.2020.1789269. 10.3109/13668250.2020.1789269 39818548

[40] Hegde S, Ellajosyula R. Capacity issues and decision-making in dementia. Ann Indian Acad Neurol. 2016;19(Suppl 1):S34–9. 27891023 10.4103/0972-2327.192890 PMC5109759

[41] Keene AR, Ward AD. With and without ‘Best interests’: the mental capacity act 2005, the adults with incapacity (Scotland) act 2000 and constructing decisions. Int J Ment Health Capacity Law. 2016;22:17–37. 10.19164/ijmhcl.v2016i22.549.

[42] Care Quality Commission. The state of health care and adult social care in England 2022/23. Newcastle upon Tyne: CQC; 2023.

[43] Stokes L, Maden M, Williams N, Jacob N, Scott S, Shepherd V, et al. Barriers and facilitators to implementation of mental capacity legislation in care homes for older adults in the united kingdom: a mixed-methods systematic review. Age Ageing. 2025;54(5):afaf119. 10.1093/ageing/afaf119. 40370080 10.1093/ageing/afaf119 PMC12078770

[44] Egede-Nissen V, Sellevold GS, Jakobsen R, Sørlie V. Minority healthcare providers experience challenges, trust, and interdependency in a multicultural team. Nurs Ethics. 2019;26(5):1326–36. 10.1177/0969733017752546. 29575974 10.1177/0969733017752546

[45] Betancourt JR, Green AR, Carrillo JE, Park ER. Cultural competence and health care disparities: key perspectives and trends. Health Aff. 2005;24(2):499–505. 10.1377/hlthaff.24.2.499. 10.1377/hlthaff.24.2.499 15757936

