# Supplementary material for: What is known about the design, delivery and implementation of mental capacity training in care homes?: a scoping review
Source: BMC Geriatr. 2025 Nov 7;25:860. doi: 10.1186/s12877-025-06476-6 (PMC12595733; doi:10.1186/s12877-025-06476-6)
Supplement: Supplementary file 1 — Supplementary Material 1. [file 12877_2025_6476_MOESM1_ESM.docx]

**Search Strategies**

Ovid MEDLINE(R) ALL <1946 to November 27, 2023>

1 exp Mental Competency/

2 "mental capacity".ti,ab,kw.

3 "mental incapacity".ti,ab,kw.

4 "adults with incapacity".ti,ab,kw.

5 "mental competenc*".ti,ab,kw.

6 "mental incompetenc*".ti,ab,kw. or deprivation of liberty safeguards.ti,ab,kw.

7 or/1-6

8 exp Nursing Homes/

9 Residential Facilities/

10 Homes for the Aged/

11 (care adj3 home*).ti,ab. or "care home*".kw.

12 (nursing adj3 home*).ti,ab. or "nursing home*".kw.

13 (residential adj3 home*).ti,ab. or "residential home*".kw.

14 "residential care".ti,ab,kw.

15 "residential facilit*".ti,ab,kw.

16 "aged care home*".ti,ab,kw.

17 "aged care facilit*".ti,ab,kw.

18 exp social support/

19 "social care".ti,ab,kw.

20 "long term care".ti,ab,kw.

21 "long term nursing care".ti,ab,kw.

22 "long term residential care".ti,ab,kw.

23 "rest home*".ti,ab,kw.

24 "institutionalized elderly".ti,ab,kw.

25 "institutionalised elderly".ti,ab,kw.

26 "extended care facilit*".ti,ab,kw.

27 "convalescen* home*".ti,ab,kw.

28 or/8-27

29 7 and 28

30 limit 29 to (yr="2000 -Current" and (english or welsh))

**CINAHL EBSCOhost**

S1 "mental capacity"

S2 "mental incapacity"

S3 "adults with incapacity"

S4 "mental competenc*"

S5 "mental incompetenc*" or "deprivation of liberty safeguards"

S6 S1 OR S2 OR S3 OR S4 OR S5

S7 (MH "Nursing Homes+") OR (MH "Residential Care+")

S8 (MH "Nursing Home Personnel") OR (MH "Nursing Home Patients")

S9 care W3 home*

S10 nursing W3 home*

S11 residential W3 home*

S12 "residential care"

S13 "residential facilit∗"

S14 "aged care home*"

S15 "aged care facilit*"

S16 "long term care"

S17 "long term nursing care"

S18 "long term residential care"

S19 "rest home*"

S20 "institutionalized elderly"

S21 "institutionalised elderly"

S22 "extended care facilit∗"

S23 "convalescence home∗"

S24 (MH "Support, Social+")

S25 "social care"

S26 S7 OR S8 OR S9 OR S10 OR S11 OR S12 OR S13 OR S14 OR S15 OR S16 OR S17 OR S18 OR S19 OR S20 OR S21 OR S22 OR S23 OR S24 OR S25

S27 S6 AND S26

S28 S6 AND S26

Limiters - Publication Year: 2000-2023

**APA PsycInfo EBSCOhost**

S1 "mental capacity"

S2 "mental incapacity"

S3 "adults with incapacity"

S4 "mental competenc*"

S5 "mental incompetenc*"

S6 S1 OR S2 OR S3 OR S4 OR S5

S7 DE "Nursing Homes" OR DE "Nursing Home Residents"

S8 care W3 home*

S9 nursing W3 home*

S10 residential W3 home*

S11 "residential care"

S12 "residential facilit*"

S13 "aged care home*"

S14 "aged care facilit*"

S15 "long term care"

S16 "long term nursing care"

S17 "long term residential care"

S18 "rest home*"

S19 "institutionalized elderly"

S20 "institutionalised elderly"

S21 "extended care facilit*"

S22 "convalescen* home*"

S23 DE "Social Support" OR DE "Perceived Social Support"

S24 "social care"

S25 S7 OR S8 OR S9 OR S10 OR S11 OR S12 OR S13 OR S14 OR S15 OR S16 OR S17 OR S18 OR S19 OR S20 OR S21 OR S22 OR S23 OR S24

S26 S6 AND S25 Limiters - Published: 20000101-20231231

**Embase**

Ovid Embase <1974 to 2023 November 27>

1 mental capacity/

2 "mental capacity".ti,ab,kw.

3 "mental incapacity".ti,ab,kw.

4 "adults with incapacity".ti,ab,kw.

5 "mental competenc*".ti,ab,kw.

6 "mental incompetenc*".ti,ab,kw. or deprivation of liberty safeguards.ti,ab,kw.

7 1 or 2 or 3 or 4 or 5 or 6

8 exp nursing home/

9 exp residential home/

10 exp home for the aged/

11 exp nursing home personnel/

12 (care adj3 home*).ti,ab. or "care home*".kw.

13 (nursing adj3 home*).ti,ab. or "nursing home*".kw.

14 (residential adj3 home*).ti,ab. or "residential home*".kw.

15 "residential care".ti,ab,kw.

16 "residential facilit*".ti,ab,kw.

17 "aged care home*".ti,ab,kw.

18 "aged care facilit*".ti,ab,kw.

19 exp social support/

20 "social care".ti,ab,kw.

21 "long term care".ti,ab,kw.

22 "long term nursing care".ti,ab,kw.

23 "long term residential care".ti,ab,kw.

24 "rest home*".ti,ab,kw.

25 "institutionalized elderly".ti,ab,kw.

26 "institutionalised elderly".ti,ab,kw.

27 "extended care facility*".ti,ab,kw.

28 "convalescen* home*".ti,ab,kw.

29 or/8-28

30 7 and 29

31 limit 30 to (english and yr="2000 - 2023")

**Social Policy and Practice Ovid <202310>**

1 "mental capacity".ti,ab.

2 "mental incapacity".ti,ab.

3 "adults with incapacity".ti,ab.

4 "mental competenc*".ti,ab.

5 "mental incompetenc*".ti,ab. or deprivation of liberty safeguards.ti,ab,kw.

6 1 or 2 or 3 or 4 or 5

7 (care adj3 home*).ti,ab.

8 (nursing adj3 home*).ti,ab.

9 (residential adj3 home*).ti,ab.

10 "residential care".ti,ab.

11 "residential facilit*".ti,ab.

12 "aged care home*".ti,ab.

13 "aged care facilit*".ti,ab.

14 "social care".ti,ab.

15 "long term care".ti,ab.

16 "long term nursing care".ti,ab.

17 "long term residential care".ti,ab.

18 "rest home*".ti,ab.

19 "institutionalized elderly".ti,ab.

20 "institutionalised elderly".ti,ab.

21 "extended care facilit*".ti,ab.

22 "convalescen* home*".ti,ab.

23 or/7-22

24 6 and 23

25 limit 24 to yr="2000 - 2023"

**HMIC Health Management Information Consortium Ovid <1979 to September 2023>**

1 exp Mental capacity/

2 "mental capacity".ti,ab.

3 "mental incapacity".ti,ab.

4 "adults with incapacity".ti,ab.

5 "mental competenc*".ti,ab.

6 "mental incompetenc*".ti,ab. or deprivation of liberty safeguards.ti,ab,kw.

7 1 or 2 or 3 or 4 or 5 or 6

8 exp Nursing homes/

9 exp Residential care/

10 exp Old peoples homes/

11 (care adj3 home*).ti,ab.

12 (nursing adj3 home*).ti,ab.

13 (residential adj3 home*).ti,ab.

14 "residential care".ti,ab.

15 "residential facilit*".ti,ab.

16 "aged care home*".ti,ab.

17 "aged care facilit*".ti,ab.

18 exp Social support/

19 "social care".ti,ab.

20 "long term care".ti,ab.

21 "long term nursing care".ti,ab.

22 "long term residential care".ti,ab.

23 "rest home*".ti,ab.

24 "institutionalized elderly".ti,ab.

25 "institutionalised elderly".ti,ab.

26 "extended care facilit*".ti,ab.

27 "convalescen* home*".ti,ab.

28 or/8-27

29 7 and 28

30 limit 29 to yr="2000 - 2023"

**Scopus**

( TITLE-ABS-KEY ( "mental capacity" OR "mental incapacity" OR "adults with incapacity" OR "mental competen*" OR "mental incompeten*" OR deprivation of liberty safeguards) AND TITLE-ABS-KEY ( ( care PRE/3 home* ) OR ( nursing PRE/3 home* ) OR ( residential PRE/3 home* ) OR "residential care" OR "residential facilit*" OR "aged care home*" OR "aged care facilit*" OR "social care" OR "long term care" OR "long term nursing care" OR "long term residential care" OR "rest home*" OR "institutionalized elderly" OR "institutionalised elderly" OR "extended care facilit*" OR "convalescen* home*" ) ) AND PUBYEAR > 1999 AND ( LIMIT-TO ( LANGUAGE , "English" ) ) AND ( LIMIT-TO ( AFFILCOUNTRY , "United Kingdom" ) )

**LENS.org**

Title/Abstract/Keyword

("mental capacity" OR "mental incapacity" OR "adults with incapacity" OR "mental competen*" OR "mental incompeten*" OR deprivation of liberty safeguards) AND ("care home* " OR "nursing home*" OR "residential home*" OR "residential care" OR "residential facilit*" OR "aged care home*" OR "aged care facilit*" OR "social care" OR "long term care" OR "long term nursing care" OR "long term residential care" OR "rest home*" OR "institutionalized elderly" OR "institutionalised elderly" OR "extended care facilit*" OR "convalescen* home*")

Limit to UK and 2000-

**Google Scholar**

A targeted search was undertaken in Google Scholar using key main search terms within the title fields only. These searches were considered supplementary to the main database searches as Google Scholar may identify relevant studies that have recently been published but not necessarily indexed in the databases.

allintitle: "mental capacity" "care home" 3 results

allintitle: "mental capacity" "care homes" 8 results

allintitle: "mental capacity" "nursing home" 1 result

allintitle: "mental capacity" "nursing homes" 3 results

allintitle: "mental capacity" "residential home" 0 results

allintitle: "mental capacity" "residential homes" 0 results

**NIHR Journals**

<https://www.journalslibrary.nihr.ac.uk/>

“mental capacity”

**NICE website**

decision-making and mental capacity Filter to Guidance AND Quality Standards

**PROQUEST**

title("mental capacity" OR "mental incapacity" OR "adults with incapacity" OR "mental competenc*" OR "mental incompetenc*" OR deprivation of liberty safeguards) AND title("care home* " OR "nursing home*" OR "residential home*" OR "residential care" OR "residential facilit*" OR "aged care home*" OR "aged care facilit*" OR "social care" OR "long term care" OR "long term nursing care" OR "long term residential care" OR "rest home*" OR "institutionalized elderly" OR "institutionalised elderly" OR "extended care facilit*" OR "convalescen* home*")

OR

title("mental capacity act") AND (MAINSUBJECT.EXACT("Nursing homes") OR "care home* " OR "nursing home*" OR "residential home*" OR "residential care" OR "residential facilit*" OR "aged care home*" OR "aged care facilit*" OR "social care" OR "long term care" OR "long term nursing care" OR "long term residential care" OR "rest home*" OR "institutionalized elderly" OR "institutionalised elderly" OR "extended care facilit*" OR "convalescen* home*")

**National Grey Literature Collection**

mental capacity act
